# The structure and toxicity of winter cyanobacterial bloom in a eutrophic lake of the temperate zone

**DOI:** 10.1007/s10646-018-1957-x

**Published:** 2018-06-22

**Authors:** Łukasz Wejnerowski, Piotr Rzymski, Mikołaj Kokociński, Jussi Meriluoto

**Affiliations:** 1 Department of Hydrobiology, Faculty of Biology, Adam Mickiewicz University, Umultowska 89, 61-614 Poznań, Poland; 2 Department of Environmental Medicine, Poznan University of Medical Sciences, Rokietnicka 8, 60-806 Poznań, Poland; 3 Biochemistry, Faculty of Science and Engineering, Åbo Akademi University, Tykistökatu 6A, 20520 Turku, Finland

**Keywords:** *Aphanizomenon gracile*, Cyanotoxins, *Planktothrix agardhii*, Toxicity, Winter cyanobacterial blooms

## Abstract

Winter cyanobacterial blooms have become increasingly common in eutrophic lakes advocating a need for their monitoring and risk assessment. The present study evaluated the toxicity of a winter cyanobacterial bloom in a eutrophicated freshwater lake located in Western Poland. The bloom was dominated by potentially toxic species: *Planktothrix agardhii*, *Limnothrix redekei*, and *Aphanizomenon gracile*. The toxin analysis revealed the presence of demethylated forms of microcystin-RR and microcystin-LR in ranges of 24.6–28.7 and 6.6–7.6 µg/L, respectively. The toxicity of sampled water was further evaluated in platelet-rich plasma isolated from healthy human subjects using lipid peroxidation and lactate dehydrogenase assays. No significant adverse effects were observed. The present study demonstrates that toxicity of some winter cyanobacterial blooms in the temperate zone, like that in Lubosińskie Lake, may not exhibit significant health risks despite microcystin production.

## Introduction

Cyanobacterial blooms constitute a major threat for aquatic ecosystems, their commercial and recreational use, and human health. Their incidence in various world regions results not only from the increased input of nutrients (phosphorus or/and nitrogen) but also from the global climate changes including temperature rise, elevation of carbon dioxide level or increased salinity (for more details see Beardall and Raven 2004; Visser et al. 2016). The greatest threat exhibited by these blooms, particularly in lakes and drinking-water reservoirs, include the presence of toxic metabolites that can be released by intact cells or during cell lysis. Moreover, cyanobacteria can also produce odorants (Su et al. 2015) that can disqualify use of water resources. As estimated, toxigenic species are globally responsible for approximately 75% of all cyanobacterial blooms (Maršálek et al. 2001; Bláhová et al. 2008).

In general, cyanotoxins constitute a very complex group of chemicals varying in their structures, physico-chemical properties, and exerted toxicity (Andersen et al. 1993; Sivonen and Jones 1999) that includes compounds such as hepatotoxic cyclic peptides (microcystins), cytotoxic alkaloids (cylindrospermopsins), teratogenic poly-methoxy-1-alkenes, neurotoxic alkaloids (anatoxin and saxitoxin analogues) as well as components of their cell wall, e.g. lipopolysaccharides (Poniedziałek et al. 2012; Durai et al. 2015). Some cyanotoxins can bioaccumulate in aquatic organisms, cause organ alterations (Magalhães et al. 2001), and even lead to massive mortality of zooplankton (Zimba et al. 2006; Baumann and Jüttner 2008), fish (Jewel et al. 2003; Svirčev et al. 2016) and other aquatic biota (Krienitz et al. 2003).

In temperate zone, massive cyanobacterial blooms most commonly occur during summer and autumn although they were also recorded in spring and rarely, in winter (Simeunović et al. 2005; Toporowska et al. 2010; Ma et al. 2016). It is hypothesized that their occurrence during the coldest months may be driven by global warming and subsequent increase in water temperature (Paerl and Huisman 2008; Paerl et al. 2011). Moreover, some cyanobacteria species have a wide thermal tolerance and can persist under ice cover. This includes species from *Planktothrix* genus that were reported to maintain high abundance in eutrophic lakes during winter (Rücker et al. 1997; Legnani et al. 2005; Toporowska et al. 2010). In addition, species of this genus (e.g. *P. agardhii* or *P. rubescens*) are among the most toxigenic cyanobacteria, potent producers of various toxins including anatoxin-a, microcystins, and/or saxitoxins (Pomati et al. 2003; Metcalf and Codd 2014).

Winter cyanobacterial blooms can also be toxic. Jacoby et al. (1994) found anatoxins in the winter bloom of cyanobacterium *Anabaena flos-aquae* Brébisson ex Bornet & Flauhault that occurred in Lake American in western Washington (USA). In turn, Ernst et al. (2001) observed a cytotoxic effect of microcystin-producing winter populations of *P. agardhii* (Gomont) Anagnostidis & Komárek from lake in Germany on whitefish and larvae (*Coregonus lavaretus*). This considered, it is imperative to study whether all winter cyanobacterial blooms can exhibit ecological and health threats.

In the present study, we report the winter cyanobacterial bloom in a eutrophic freshwater lake in Western Poland. As revealed in previous research, the lake is dominated by toxic *P. agardhii* during the whole year (Mankiewicz-Boczek et al. 2009; Kobos et al. 2013). We hypothesized that (i) significant concentrations of toxins, particularly microcystins, may be present in water, and (ii) the bloom will exhibit a health threat due to the occurrence of the potent secondary metabolites. Therefore, the water samples were screened for the presence of microcystins, and their toxicity was evaluated by a method employing human platelet-rich plasma (PRP) with malondialdehyde (MDA) and lactate dehydrogenase (LDH) as markers of oxidative stress and cytotoxicity, respectively. This model was selected because platelets has been reported to be severely impacted during microcystin intoxications (Stoner et al. 1989; Giannuzzi et al. 2011; Rankin et al. 2013) while cyanotoxins, including microcystins, are known to induce lipid peroxidation leading to increase in MDA content and subsequent decrease in cellular viability marked by LDH leakage (Huang et al. 2015; Rzymski et al. 2017). Our study adds to toxicity assessment of cyanobacterial blooms that may occur in temperate zone during a winter period.

## Methods

### Sampling

Water samples were collected in January 2017 from Lake Lubosińskie (52°31′40″N, 16°22′56″E) for physico-chemical analyses, phytoplankton analysis, toxins analyses, and toxicological screening. Samples were taken using bathometer (UWITEC, Austria) from a surface layer (10–50 cm) under ice-cover (10 cm). Phytoplankton samples were fixed with Lugol’s solution 1% and stored in darkness. Three sub-samples for toxin analyses were lyophilized immediately after sampling in the laboratory, and they were stored in the freezer at −20 °C. Samples for toxicity tests were filtered through 0.22 μm syringeless filter devices and frozen at −40 °C prior to analyses.

### Physico-chemical analyses

Sampled water was a subject to physico-chemical analyses. The following parameteres were analysed: pH, reduction–oxidation reaction (FiveEasy™ Benchtop Meter, Mettler Toledo, USA), electrical conductivity (Hanna Instruments, USA), dissolved oxygen (O_2_, using the Winkler method), NH_4_^+^ (using the Nessler method), nitrites (NO_2_^−^, using the sulphanic acid method), nitrates (NO_3_^−^, using the sodium salicylate method), TP (using the molybdate method after mineralization), ortophosphates (total reactive phosphorus, TRP, using the molybdate method), total iron (total Fe, using the *o*-phenanthroline method), calcium (Ca^2+^, using titration method with standardized EDTA solution), total hardness (TH, using the complexometric titration with EDTA), chloride (Cl^−^, using the mercuric nitrate method) (APHA 2005). Inorganic nitrogen was calculated as a sum of NH_4_^+^, NO_2_^−^ and NO_3_^−^; organic phosphorus (P_org._) was calculated by subtracting TRP from TP. The magnesium (Mg^2+^) concentration (mg/L) was calculated from the equation:

$${\mathrm{Mg}}^{2+} {\mathrm{concentration}} = \frac{{\left( {{\mathrm{Total}}\,{\mathrm{hardness}}\,\left( {{\mathrm{mg}}\,{\mathrm{CaCO}}_{3}/{\mathrm{L}}} \right){\mathrm{-2.497}}} \right) \times {\mathrm{Ca}}^{2+}{\mathrm{concentration}}\,\left( {{\mathrm{mg/L}}} \right)}}{{4.118}}$$

where 2.497 is the multiplying factor for Ca^2+^ as CaCO_3_ which is calculated by dividing the equivalent weight of CaCO_3_ to the equivalent weight of Ca^2+^, and 4.118 is the multiplying factor for Mg^2+^ as CaCO_3_ which is calculated by dividing the equivalent weight of CaCO_3_ to the equivalent weight of Mg^2+^ (APHA 2005).

### Phytoplankton analysis

Prior analyses, phytoplankton samples were sedimented in 1-L glass cylinder for 48 h, after which the overlying water was gently decanted off and lower layer (volume 20–40 mL) was used for phytoplankton analysis. Species identification and counts were conducted using light microscope Olympus under ×400 magnification. The phytoplankton enumeration was carried out in 100–150 fields of Fuchs-Rosenthal chamber, which ensured that at least 400 specimens were counted to reduce the error to less than 10%. A single cell, a coenobium, or a filament represented one specimen in the analysis. The biovolume of each species was determined through a volumetric analysis of cells using geometric approximation and expressed as a wet weight following Wetzel and Likens (2000).

### Toxin analyses

Lyophilized water sub-samples were used to evaluate the presence or absence of three homologues of microcystins (MC-RR, -YR, -LR) and their demethylated forms using HPLC-diode-array UV detection (HPLC-DAD) and HPLC-MS/MS analysis. Samples preparation and determination of microcystins were performed according to the previous description (Hautala et al. 2013).

### Toxicological screening

Toxicity of sampled water was assessed using a model employing human blood collected from five healthy donors at the Regional Centre of Blood and Blood Treatment in Poznan, Poland, according to accepted safeguard standards and legal requirements in Poland. Blood samples from each donor were centrifuged at 200×*g* for 12 min to obtain platelet-rich plasma (PRP), divided into three groups:

(i) Group 1 (negative control group): 1 mL of PRP without any treatments;
(ii) Group 2 (positive control group): 1 mL of PRP incubated with 10 μM of *tert*-butyl hydroperoxide (*t*-BHP), a well-established inducer of oxidative stress and specifically, lipid peroxidation (Komosa et al. 2017; Poniedziałek et al. 2017);
(iii) Group 3 (treatment group): 1 mL of PRP incubated with 100 µL of water sample filtered through 0.22 μm syringeless filter devices.

All samples were incubated at 37 °C for 1 h. Following the incubation, oxidative stress and cytotoxicity were assessed. The former was based on concentration of MDA, a major product of lipid peroxidation (Ayala et al. 2014). Along with 4-hydroxy-2-nonenal, MDA is a major final product of this process, classically measured to assess the level of oxidative damage triggered by different toxins. Cells undergoing death release LDH rapidly to the extracellular environment, in which it remains relatively stable. For this reason, LDH is one of the most widely used markers of cytotoxicity (Fotakis and Timbrell 2006), and was also applied in our toxicological evaluation.

After incubation all samples were mixed on ice with 300 μL of RIPA Buffer (50 mM Tris-HCl, pH 7.4, 1% Triton X-100, 150 mM NaCl, 1% Tergitol-type NP-40, 0.5% sodium deoxycholate, 0.1% sodium dodecyl sulfate) to conduct the lysis of cellular component. RIPA buffer was supplemented with butylated hydroxytoluene (BHT) to prevent artificial lipid peroxidation. The samples were then centrifuged (1600×*g*, 10 min, 4 °C) to remove insoluble material. The collected supernatants were used to determine the MDA using TBARS Assay Kit (Cayman Chemical, USA). Briefly, 100 µL of each supernatant was mixed with 100 µL of 10% C_2_HCl_3_O_2_ and 800 µL of working reagent: a mixture of thiobarbituric acid (TBA), acetic acid and NaOH. The samples were then boiled for 1 h to accelerate formation of MDA-TBA adduct and the reaction was terminated by incubation on ice for 10 min. After centrifugation (4500 rpm, 4 °C, 10 min), 200 µL of supernatant (in duplicate) was transferred to 96-well plate. The absorbance of the product was read at 532 nm. The calculated values were compared to a calibration curve prepared using MDA standard (Cayman Chemical, USA; *r*^2^ = 0.99). results were expressed as µM of MDA.

Cytotoxicity was assessed using Cytotoxicity Detection LDH Kit (Sigma-Aldrich,Germany) according to the manufacturer’s instructions. Following the incubation, all samples were centrifuged for 10 min at 1000 rpm and supernatants (100 µL) were transferred to 96-well flat bottom microplate and mixed with 100 µL reaction cocktail containing iodotetrazolium chloride, sodium lactate and diaphorase/NAD+ mixture. After incubation (30 min, 25 °C, darkness), the absorbance of each sample was read at 492 nm, and cytotoxicity of lake water sample was calculated according to equation:

$${\mathrm{Cytotoxicity}}\,\left[ {\mathrm{\% }} \right] = \frac{{{\mathrm{Absorbance}}\,\left( {{\mathrm{sample}}} \right){\mathrm{ - Absorbance}}\,\left( {{\mathrm{control}}} \right)}}{{{\mathrm{Absorbance}}\,\left( {{\mathrm{high}}\,{\mathrm{control}}} \right){\mathrm{ - Absorbance}}\,\left( {{\mathrm{control}}} \right)}} \times 100$$

where control is a non-exposed sample and high control represents a non-exposed sample mixed with RIPA lysis buffer to evaluate the maximum releasable LDH activity for each sample.

## RESULTS

### Physico-chemical analysis

Physico-chemical analysis showed high concentrations of total phosphorus and ammonium indicating high trophic level of Lake Lubosińskie. Also chlorophyll-*a* concentration, pH and conductivity values confirmed eutrophic character of this ecosystem. The physico-chemical parameters and chlorophyll-*a* concentration of investigated lake are given in Table 1.

**Table 1:** The physico-chemical parameters of water sampled and chlorophyll-*a* concentration during winter cyanobacterial bloom at Lubosińskie Lake

Parameter	Measurement
pH	8.12
Reduction–oxidation reaction [mV]	−63
Temperature [°C]	3
Electrical conductivity [µS/cm]	511
O_2_ [mg/L]	5.5
TP [mg/L]	0.025
TRP [mg/L]	0.0085
P_org._ [mg/L]	0.0165
NH_4_^+^ [mg/L]	0.92
NO_2_^−^ [mg/L]	0.005
NO_3_^−^ [mg/L]	0.025
N_inorg._ [mg/L]	0.95
Cl^−^ [mg/L]	60
Ca^2+^ [mg/L]	47
Mg^2+^ [mg/L]	19.0
Total Fe [mg/L]	0.01
Total hardness [mg CaCO_3_/L]	195.8
Chlorophyll-*a* [µg/L]	61.01

### Phytoplankton analysis

**Fig. 1:**
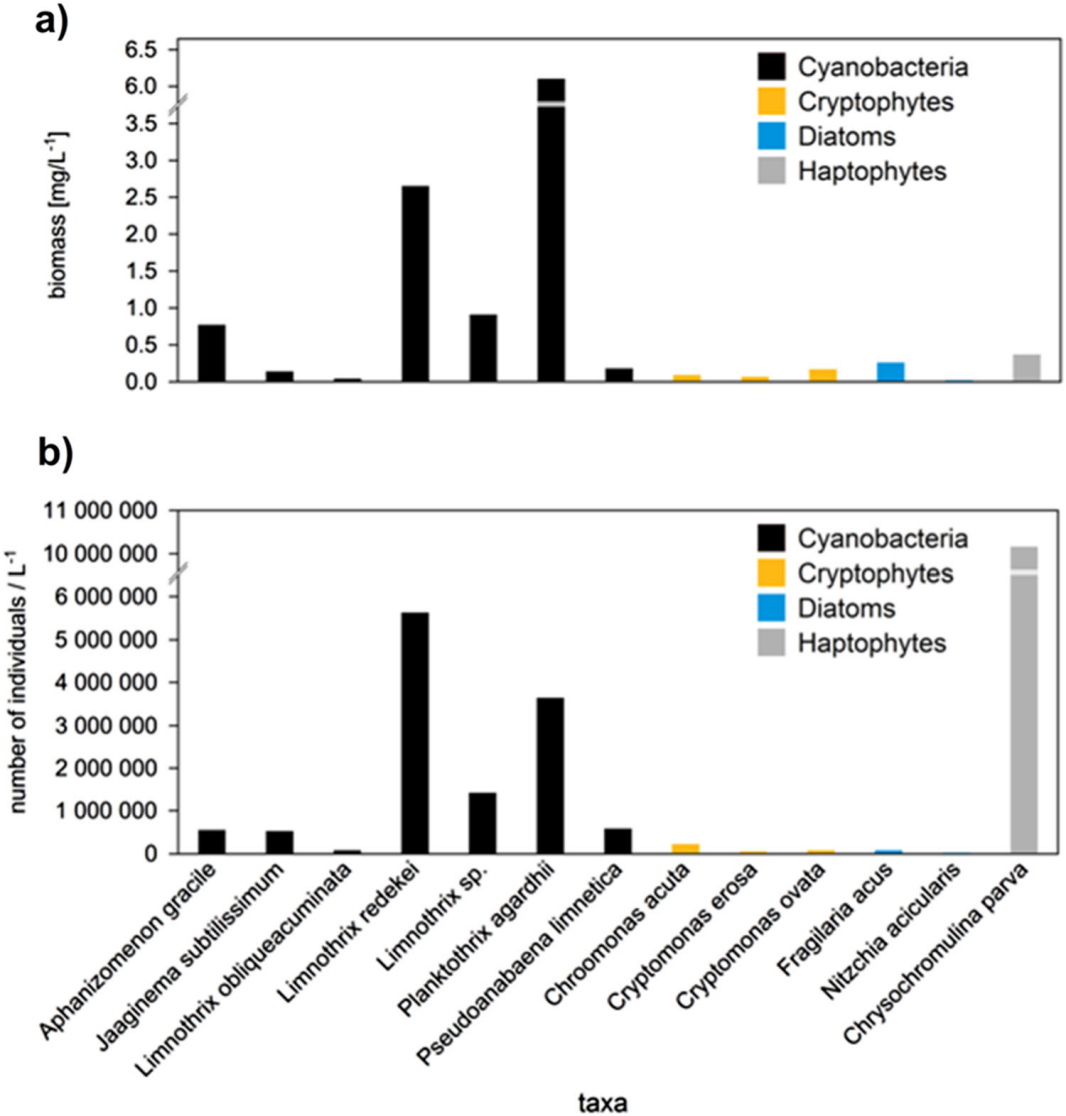
Phytoplankton composition in Lubosińskie Lake during winter 2017: the biomass of organisms (**a**) and their abundance (**b**)

Total phytoplankton biomass exceeded 11 mg/L indicating eutrophic status of the lake. Phytoplankton community was dominated by cyanobacteria, which accounted for over 90% of total phytoplankton biomass (Fig. 1). *P. agardhii* was the most abundant species contributing over 50% to the total algae biomass. Other filamentous cyanobacteria, *Limnothrix redekei* and *Aphanizomenon gracile*, were also common species, the overall contribution of both taxa exceeded 22% and 6% of the total biomass, respectively. Other groups of algae were less abundant with the most numerous haptophyte *Chrysochromulina parva* (Fig. 1).

### Toxin analyses

**Fig. 2:**
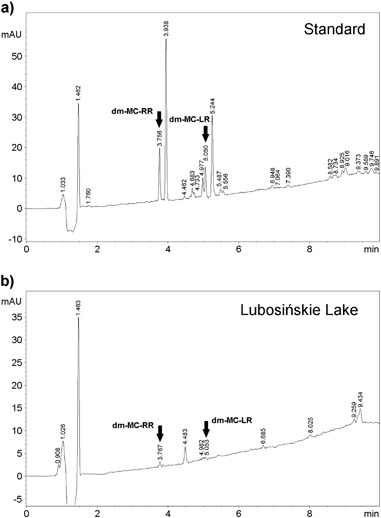
The results of HPLC-DAD analysis: HPLC-UV chromatograms of MCs standard (**a**) and sample from Lubosińskie Lake (**b**)

**Fig. 3:**
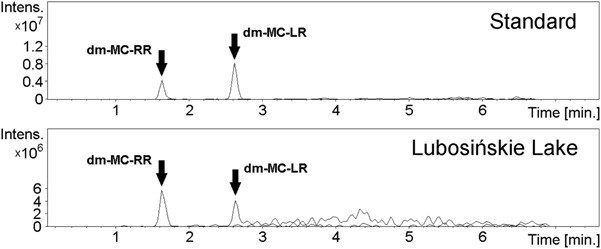
The results of HPLC-MS/MS analysis: extracted ion chromatograms of *m*/*z* 512.9 (dm-MC-RR) and *m*/*z* 981.6 (dm-MC-LR) of MCs standard and sample from Lubosińskie Lake

Both HPLC-DAD and HPLC-MS/MS revealed the absence of MC-RR, -YR, and -LR but the demethylated forms of MC-RR and MC-LR were present in each analysed water sub-sample from Lake Lubosińskie (Figs. 2 and 3). The concentration of demethylated MC-RR and demethylated MC-LR in lake water ranged from 24.6 to 28.7 µg/L and 6.6 to 7.6 µg/L, respectively.

### Toxicological screening

**Fig. 4:**
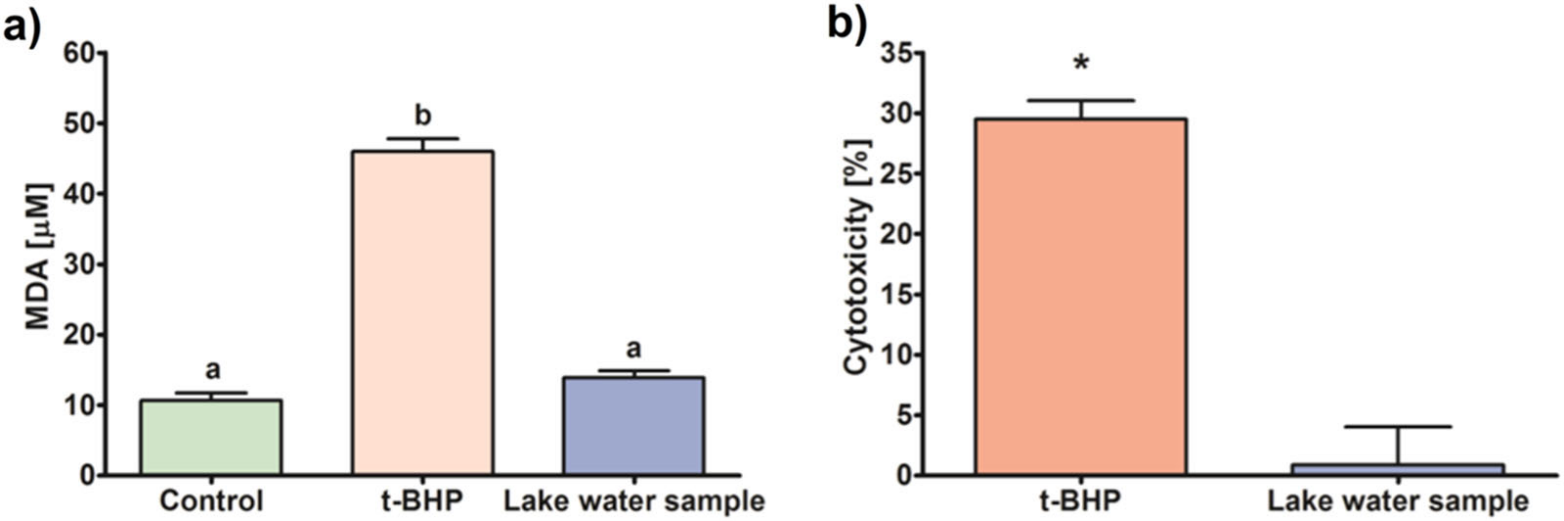
The toxicological assessment of the filtered water from Lake Lubosińskie sampled during winter cyanobacterial bloom. Effects were measured in the human platelet-rich plasma (PRP) and compared to those exerted by *tert*-butyl hydroperoxide (*t*-BHP; positive control). **(a)** The malondialdehyde (MDA) concentration (mean ± SD) as measured by TBARS assay (*n* = 5). Different superscript letters denote statistically significant difference (*p* < 0.05) according to Tukey’s HSD test (MANOVA). **(b)** The cytotoxicity (mean ± SD) evaluated by means of lactate dehydrogenase (LDH) leakage. Asterisk indicates statistically significant difference with control (*p* < 0.05; Student *t*-test)

The mean ± standard deviation content of MDA in human PRP treated with water samples (13.9 ± 1.7 µM) was only slightly and insignificantly increased compared to untreated control (10.7 ± 1.8 µM) supporting that no oxidative damage was induced despite relatively high volume of lake water (1:10, v-v) added to PRP. This was further confirmed by no significant cytotoxicity (0.9 ± 6.3 %) as measured by LDH leakage assay (Fig. 4).

## Discussion

The present study demonstrates that toxicity of some winter cyanobacterial blooms in the temperate zone may not exhibit significant health risks even though this could be expected from the microcystin production. In the studied Lubosińskie Lake, the winter phytoplankton biomass was dominated by filamentous cyanobacteria including potentially toxic species *P. agardhii*, *L. redekei*, and *A. gracile*. Total biomass of all identified cyanobacteria species exceeded 90% of the total phytoplankton biomass indicating a hypertrophic status of the lake and the presence of winter cyanobaterial bloom. Moreover, chlorophyll-*a* and nutrient concentration in the water indicated a high trophic status of the studied lake. Cyanobacterium *P. agardhii*, a common producer of microcystins (Fastner et al. 1999) contributed over 50% to the total phytoplankton biomass. Earlier study have showed perennial toxic blooms in this lake (Kokociński et al. 2009; Mankiewicz-Boczek et al. 2009; Kobos et al. 2013), and demethylated forms of microcystin-LR and microcystin-RR were found in this study. Numerous studies have shown that toxin production in cyanobacteria is slowed down at temperatures exceeding the optimal ranges (Codd and Ponn 1988; Lehtimäki et al. 1994; Rapala and Sivonen 1998). This is also supported by polar research performed by Kleinteich et al. (2012), which demonstrated that cyanobacterial diversity in the polar mats, share of toxin-producing cyanobacteria and cyanotoxins concentration increase over the temperature gradient.

The next part of our work was focused on the assessment of the toxicity of sampled water from Lake Lubosińskie using a model employing human blood. The use of human PRP derived from blood of healthy donors, and use of MDA and LDH as markers can be considered as a convenient and rapid method to evaluate toxicity of studied samples. It does not require complicated isolation steps/culturing, and responses are recorded in a rapid manner in the presence of some cellular and extracellular components of blood. This model provides a simplicity over specificity as an exact mechanism of action and target cells cannot be established using this method. However it can be very useful in general screening for toxicity. Various cyanotoxins, including microcystins can induce oxidative stress and increase cellular content of MDA (Moreno et al. 2005; Rzymski et al. 2017). Based on this method, lake water samples were not found to exert any toxicity in employed *in vitro* model, indicating that the demethylated forms of microcystins at determined concentrations do not pose any major health threat.

Winter cyanobacterial blooms become more common phenomenon in lakes, what can be inferred from the increasing number of reports from the field surveys (Skulberg 1964; Willén and Mattsson 1997; Rücker et al. 1997; Ernst et al. 2001; Legnani et al. 2005; Simeunović et al. 2005; Toporowska et al. 2010; Babanazarova et al. 2013; Ma et al. 2016). Most common overwintering cyanobacteria in lakes are species of the genera *Planktothrix* and *Limnothrix*. Toporowska et al. (2010) found that *P. agardhii* filaments increase in length during autumn and it was interpreted as an adaptation to overwinter. On the other hand, a dominance of *P. agardhii* in lakes during winter may also be due to availability of ammonium, which occurs often in higher concentration during winter in lakes (Toporowska et al. 2010). This concept can be supported by the results of Donald et al. (2011), which revealed that availability of ammonium can favour non-heterocytous cyanobacteria over N_2_-fixers like nostocaleans. In support of this, in the present study we noted a relatively high ammonium concentration (0.92 mg/L). Furthermore, *P. agardhii* possesses a higher resistance against potential grazing zooplankton in comparison to number of other cyanobacteria associated with often distinctly wider filaments, covered on the surface by mucilaginous sheaths (Komárek and Komárková 2004; Wejnerowski et al. 2018).

A unique features of cyanobacteria, e.g. relatively low light and nutrient requirements (Tilzer 1987; Reynolds and Walsby 1975; respectively), wide thermal tolerance (Robarts and Zohary 1987), buoyancy regulation (Reynolds et al. 1987) allow them to dominate the lake phytoplankton. High temperature, however, is frequently considered as a key factor determining the dominance and further expansion of cyanobacterial blooms (Paerl and Huisman 2008; Paerl et al. 2011). This is due to their optimum growth rates at higher temperature (Butterwick et al. 2005; Helbling et al. 2015). Moreover cyanobacteria show greater affinity for nutrients at elevated temperatures compared to eukaryotic algae (Xie et al. 2012). This study showed nonetheless, that some cyanobacteria including *P. agardhii* may benefit from low temperatures and sustain abundant population even beneath ice cover. Dominance of this cyanobacterium during low temperature, however, was not correlated with high toxins concentration, what is in line with recent findings of Walls et al. (2018). Therefore, it seems that winter blooms may pose serious threats for aquatic ecosystem due to high biomass production and phytoplankton community alteration but not always due to toxin production.

## Conclusions

The present study reports a winter cyanobacterial bloom dominated by potentially toxic cyanobacteria, *P. agardhii*, *L. redekei*, and *A. gracile*. HPLC analyses revealed the presence of demethylated forms of MC-RR and MC-LR in the sampled lake water. Toxicological screening performed on sampled water using *in vitro* human cell model revealed that at these concentrations detected toxins are not likely to pose a health threat.
